# Maternal prenatal urinary bisphenol A level and child cardio-metabolic risk factors: A prospective cohort study^☆^

**DOI:** 10.1016/j.envpol.2020.115008

**Published:** 2020-10

**Authors:** Fengxiu Ouyang, Guang-Hui Zhang, Kun Du, Lixiao Shen, Rui Ma, Xia Wang, Xiaobin Wang, Jun Zhang

**Affiliations:** a Ministry of Education and Shanghai Key Laboratory of Children’s Environmental Health, Xinhua Hospital, Shanghai Jiao Tong University School of Medicine, Shanghai, China; b Department of Clinical Laboratory Test, Xinhua Hospital, Shanghai Jiao Tong University School of Medicine, Shanghai, China; c Center on the Early Life Origins of Disease, Department of Population, Family and Reproductive Health, Johns Hopkins University Bloomberg School of Public Health, Johns Hopkins University School of Medicine, Baltimore, MD, USA

**Keywords:** Prenatal BPA, Child blood pressure, Child cardio-metabolic risk factors, Birth cohort

## Abstract

Exposure to endocrine disrupting chemicals during the first 1000 days of life may have long-lasting adverse effects on cardio-metabolic risk in later life. This study aimed to examine the associations between maternal prenatal Bisphenol A (BPA) exposure and child cardio-metabolic risk factors at age 2 years in a prospective cohort. During 2012–2013, 218 pregnant women were enrolled at late pregnancy from Shanghai, China. Urinary BPA concentration was measured in prenatal and child 2-year spot urine samples, and classified into high, medium and low tertiles. Child adiposity anthropometric measurements, random morning plasma glucose, serum insulin, and lipids (high-density lipoprotein, low-density lipoprotein, cholesterol, triglyceride), systolic (SBP) and diastolic blood pressure (DBP) were measured. Linear regression was used to evaluate the associations between prenatal BPA and each of the cardio-metabolic risk factors in boys and girls, respectively, adjusting for pertinent prenatal, perinatal and postnatal factors. BPA was detectable (>0.1 μg/L) in 98.2% of mothers prenatally and 99.4% of children at age 2 years. Compared to those with low prenatal BPA, mean SBP was 7.0 (95%CI: 2.9–11.2) mmHg higher, and DBP was 4.4 (95%CI: 1.2–7.5) mmHg higher in girls with high prenatal BPA levels, but these associations were not found in boys. In boys, medium maternal prenatal BPA level was associated with 0.36 (95% CI: 0.04–0.68) mmol/L higher plasma glucose. No associations were found between prenatal BPA and child BMI, skinfold thicknesses, serum lipids, or insulin in either girls or boys. There were no associations between concurrent child urinary BPA and cardio-metabolic risk factors. These results support that BPA exposure during prenatal period, susceptible time for fetal development, may be associated with increase in child BP and plasma glucose in a sex-specific manner. Further independent cohort studies are needed to confirm the findings.

## Highlights

• Maternal prenatal BPA was associated sex-specifically with child blood pressure.
• Prenatal BPA was associated with higher blood pressure in girls at 2 years.
• Medium prenatal BPA was associated with higher plasma glucose in boys.
• No associations between concurrent child BPA and cardio-metabolic risk factors.

## Graphical abstract

**Figure:**
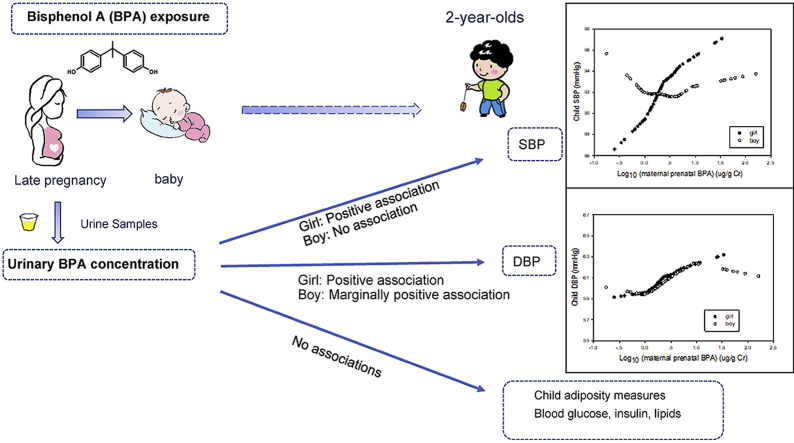

## 1 Introduction

Bisphenol A (BPA) is one of the most common environmental endocrine disrupting chemicals (EDCs). It has been extensively used as an additive/plasticizer in epoxy resins and polycarbonate (PC) plastic for more than 40 years, which has resulted in the wide use of BPA in various daily-related products including plastic food containers, water bottles, food cans (in the inner coating of metal), water pipeline (linings inside), thermal printing paper, and dental sealants(Huang et al., 2012). BPA is ubiquitous in the environment, and has been detected in more than 90–95% of populations worldwide, including pregnant women (Wang et al., 2017). Maternal BPA can easily cross the placenta and reach fetus, which is evidenced by the detection of BPA in the cord blood (Lee et al., 2018).

BPA is a typical EDC due to its estrogenic activities and its potential for endocrine disruption including thyroid-hormone disrupting (Park et al., 2020; Zhang et al., 2018). EDC exposure during the fetal period, a window of high susceptibility that is critical for cardiovascular system development, may alter developmental programing, leading to lifelong effects including cardiovascular diseases and its precursors (Kamai et al., 2019; Mone et al., 2004; Sanders et al., 2018). In a rodent model, oral administration of BPA increased both systolic blood pressure (SBP) and diastolic blood pressure (DBP) (Saura et al., 2014). Toxicological studies found that BPA disrupted cardiovascular physiology by interfering with endogenous hormones (Acconcia et al., 2015). Exposure to EDCs during the first 1000 days of life may have long-lasting effects on cardio-metabolic health in later life.

Cardiovascular diseases (CVDs) have become an important cause of premature death (World Health Organization, 2011). Hypertension has high prevalence worldwide, and is the major preventable cause of strokes (Chow et al., 2013). Overall, hypertension prevalence was about 30–45% in adults (NCD Risk Factor Collaboration (NCD-RisC), 2017), with a global age-standardized prevalence of 24% in men and 20% in women in 2015. High blood pressure (BP) in childhood predicts increased risk of hypertension and cardiovascular events in later life (Lurbe and Ingelfinger, 2016; Oikonen et al., 2016). Few studies have examined maternal prenatal BPA exposure and child cardio-metabolic risk factors, and the three existing studies have reported inconsistent findings (Bae et al., 2017; Vafeiadi et al., 2016; Warembourg et al., 2019). A prospective study conducted in South Korea reported a positive association of prenatal urinary BPA concentration with DBP in boys, and SBP in girls at age 4 years with BPA above certain threshold, and a negative prenatal-BPA and SBP association in girls with BPA below the threshold (Bae et al., 2017). A similar prospective study in Greece reported that prenatal urinary BPA was associated with lower BMI and adiposity measures in girls (assessed at 2.5 and 4 years of age) and increase of such factors in boys, but no associations with child cardiometabolic risk factors (BP, total cholesterol or high-density lipoprotein (HDL) cholesterol) were observed in children aged 4 years (Vafeiadi et al., 2016). In a recent analysis of data from the European Human Early-Life Exposome (HELIX) cohort, gestational BPA exposure was associated with increases in DBP and, to a lesser extent, with higher SBP in children (Warembourg et al., 2019). None of the three studies found an association between concurrent child BPA and BP (Bae et al., 2017; Vafeiadi et al., 2016; Warembourg et al., 2019).

In this study, we aimed to examine the associations between prenatal BPA exposure and child adiposity measures, BP and other metabolic risk factors (blood glucose/insulin, serum lipids) in boys and girls at age 2 years.

## 2 Methods

### 2.1 Study population

This report used data collected from mothers and children enrolled in the Shanghai Obesity and Allergy Birth Cohort Study. The baseline study was implemented between June 2012 and February 2013 at a large tertiary maternity and child health hospital in Shanghai, China where women were enrolled as they were admitted to hospital for childbirth. Eligibility criteria included: 1) singleton pregnancy; 2) receiving routine prenatal care; 3) plan to stay Shanghai at least in the next 2 years; and 4) willing to signed informed consent and participate in this study. After enrollment, a face-to-face maternal questionnaire interview was conducted by trained research nurses and spot urine samples of study women were collected. After delivery, the study nurses reviewed medical records of maternal prenatal care, laboratory reports and delivery information to obtain clinical data, of pregnancy complications and birth outcomes (gestational age at delivery, infant sex, birthweight, and birth length) using a standardized abstraction form. The infants of the study women were invited to participate in an internet-based questionnaire investigation at age 6 months, and follow-up visits at age 1 and 2 years at Xinhua Hospital, a large tertiary hospital in Shanghai. The postnatal 2-year follow-up study included a face-to-face questionnaire interview, anthropometric and BP measures, and the child’s morning blood draw and urine samples collection. At baseline, 620 women provided urine samples and had valid prenatal BPA measures (Wang et al., 2017). At age 2 years, 218 children had completed the follow-up survey and provided both blood and urine samples as described above. This study included 218 mothers and their 2-year-olds.

All participating women provided written informed consent. The study was approved by the research ethics committees of Xinhua Hospital affiliated with Shanghai Jiao Tong University School of Medicine and the International Peace Maternity and Child Hospital.

### 2.2 Urinary BPA assessment

Urinary concentration of total BPA (i.e. free plus conjugated) was assessed by using a modified high-performance liquid chromatography-tandem mass spectrometry (HPLC-MS/MS) analytical method as described previously (Chen et al., 2012; Wang et al., 2017). The limit of detection (LOD) of BPA was 0.1 μg/L. Briefly, ammonium acetate buffer solution (pH = 5.0, 1 mmol/L) was used to dilute internal isotope and urine sample, then β-glucuronides (Type H-1 from Helix Pomatia, Sigma-Aldrich, USA) was used to de-conjugate and incubate overnight at 37 °C. Next, solid phase extraction (500 mg/3 mL; Supelco, ENVI-18, USA) was used to purify the samples. After being centrifuged by a high-speed vacuum centrifuge (Thermos.co, Thermos SPD 121 P) and drying, it was dissolved using methanol, and analyzed by a HPLC-MS/MS system (Agilent 1290–6490, USA). We prepared quality control (QC) samples from the BPA standard (Augsburg, Germany) and the pooled urine sample. Quality assurance and quality control (QA/QC) procedures has been detailed in our previous publication (Wang et al., 2017). The urinary concentrations of creatinine were measured by Enzymatic Creatinine_2 Reagents (ECRE_2) on an automated chemistry analyzer (7100 Hitachi, Tokyo, Japan).

### 2.3 Child anthropometric measurements and blood pressure

At postnatal 2-year follow-up visit, children were measured anthropometric measurements which included body length, weight, mid-upper arm circumference (MUAC) and skinfold thickness (triceps, subscapular and abdominal). Body weight was assessed to the nearest 100 g (Seca 956 Scale), body length, in a supine position to the nearest 0.1 cm (Seca 416 infantometer), MUAC to the nearest 0.1 cm with inelastic tape. Skinfold thicknesses were measured to the nearest 1 mm by our two study pediatricians with calipers (Lange, Beta Technology, Santa Cruz, California, USA). The SBP and DBP were measured on the child’s left arm, at least 30 min after the child arrived for the study visit, by a mercury sphygmomanometer and an appropriate size cuff for arm circumference. Three readings were taken 1 min apart and the average was used in the analysis. Child length and weight were measured by two trained study nurses. Child head circumference, MUAC, skinfold thickness and blood pressure were measured by the study pediatricians.

Sex-specific weight-for-length and weight-for-age z-scores were calculated using WHO Child Growth Standards. Z-score = (observed value - median value of WHO growth standards)/standard deviation (SD) from WHO growth standard (http://www.who.int/childgrowth/standards/en/) (de Onis et al., 2012). Overweight and obesity was defined as weight-for-length > 2 SD, and >3 SD above the WHO Child Growth Standards median value, respectively. Wasting was defined as weight-for-height < -2 SD of the WHO Growth Standards. Underweight is defined as weight-for-age < -2 SD from the WHO Standards.

### 2.4 Assessment of blood lipids, glucose, and insulin in children

Plasma glucose, serum lipids, and insulin were measured in the laboratory of Xinhua Hospital, which was certified by the China National Accreditation Service for Conformity Assessment. Serum HDL, low-density lipoprotein (LDL), cholesterol, triglyceride, and plasma glucose were measured by a Hitachi 917 auto-analyzer, and serum insulin was measured on a Beckman DXI 800 autoanalyzer. Parents were asked to record the breakfast time of their children.

### 2.5 Major covariates

Maternal height, hypertensive disorders in pregnancy (which included chronic hypertension, gestational hypertension, preeclampsia, and eclampsia), infant sex, gestational age and infant birthweight were obtained from the hospital medical records. The maternal age at childbirth, prepregnancy weight, education level, and information about smoking and secondhand smoke (husband smoking) were self-reported at the baseline questionnaire interview (Ouyang et al., 2018). Maternal prepregnancy BMI (kg/m^2^) was defined as prepregnancy weight (kg)/height squared (m^2^). Based on sex-specific Chinese reference standards for birthweight at each gestational week, we defined small for gestational age (SGA) as birthweight <10th percentile, appropriate for gestational age (AGA) as 10th-90th percentile, and large for gestational age (LGA) as >90th percentile, respectively (Zhu et al., 2015).

Child age was calculated as date at follow-up visit minus birth date. Infant feeding type during the first 6 months was obtained by self-report at the 6-month postnatal follow-up online investigation and was grouped into three types: (1) exclusive breastfeeding, (2) mixed feeding which is the combination of breastfeeding and formula feeding, and (3) exclusive/only formula feeding (Xu et al., 2009). Child secondhand smoking exposure was determined based on questionnaires at the 6-, 12- and 24-month postnatal investigations, and defined as mother smokes, father smokes, or anyone else who lives in the home smokes.

### 2.6 Data analysis and statistics

Maternal prenatal urinary BPA concentration was classified into the high, medium and low by tertiles. Firstly, we compared the maternal characteristics, child characteristics and child cardio-metabolic risk factors by tertile of maternal prenatal BPA. We used *χ*^2^ tests for categorical variables and ANOVA F-tests for continuous variables to test the difference among the three prenatal BPA groups (Table 1, Table 2). The analysis was performed in boys and girls separately. The locally weighted nonparametric smoothing scatter plots (SAS LOESS) was used to graphically explore the associations of maternal prenatal urinary BPA concentration in base 10 logarithm transformation with child SBP and DBP with adjustment for child age and stratified by infant sex. Then, to examine the associations between maternal prenatal urinary BPA concentration and child cardio-metabolic risk factors (adiposity measures, BP, serum lipid profiles, serum insulin, plasma glucose), multivariate linear regression models were performed with child cardio-metabolic measures as a dependent variable. All regression models included the natural logarithm of prenatal urinary creatinine concentration and the known covariates (child age) or potential risk factors for cardio-metabolic measures (birthweight for gestational age, weight-for-length z-score, maternal passive smoking, child passive smoking, infant 0–6 months breastfeeding type), and were performed in boys and girls separately. Model 1 included child age; Model 2 was additionally adjusted for birthweight for gestational age (LGA and non-LGA); Model 3 was also adjusted for child urinary BPA (low, medium and high level in tertiles) and weight-for-length z-score; Model 4 also included maternal passive smoking (yes or no), child passive smoking (yes or no), and infant 0–6 months breastfeeding type (formula, exclusive breastfeeding, and mixed feeding). The approach for selection of covariates were to control the potential confounders and gain model precision. In addition, to evaluate the changes in child cardio-metabolic variables per each 10-fold increase of prenatal BPA, the log_10_-transformed prenatal BPA was used in the models. For urinary BPA < LOD of the assay, a value equal to 0.1 μg/L (the LOD) divided by the square root of 2 was used for logarithm transformation. All the analyses were conducted with SAS 9.2 software (SAS Institute, Cary, North Carolina). The level of statistical significance used a two-sided p < 0.01.

**Table 1:** Parental and infant characteristics at baseline and 2-year postnatal visit, by infant sex and maternal prenatal urinary BPA level (low, medium, and high).

	Maternal prenatal BPA (μg/L)			p value	Maternal prenatal BPA (μg/L)			
	1st tertile (low)	2nd tertile	3rd tertile (high)		1st tertile (low)	2nd tertile	3rd tertile (high)	p value
Sample size	41	38	34		31	35	39	
	*mean* ± *SD*							
Log10 (maternal urinary BPA) (μg/L)	−0.39 ± 0.25	0.08 ± 0.11	0.80 ± 0.50	<0.001	−0.41 ± 0.30	0.08 ± 0.10	0.69 ± 0.43	<0.001
Log10 (maternal creatinine-adjusted BPA) (μg/g Cr)	0.10 ± 0.27	0.35 ± 0.26	0.89 ± 0.55	<0.001	0.07 ± 0.32	0.35 ± 0.23	0.81 ± 0.59	<0.001
Log10 (child BPA) (μg/L) at 2 years	0.004 ± 0.43	0.13 ± 0.61	0.13 ± 0.32	0.52	−0.01 ± 0.29	0.01 ± 0.35	0.05 ± 0.45	0.85
Log10 (child creatinine-adjusted BPA) (μg/g Cr)	0.49 ± 0.44	0.58 ± 0.57	0.51 ± 0.31	0.73	0.52 ± 0.34	0.45 ± 0.34	0.57 ± 0.43	0.48
**Parental Factors**								
Maternal age at childbirth (Years)	30.4 ± 2.9	30.2 ± 3.2	30.3 ± 3.3	0.99	32.0 ± 4.7	30.8 ± 3.9	30.5 ± 2.7	0.21
Pre-pregnancy BMI (kg/m^2^)	20.9 ± 2.8	21.7 ± 3.3	21.3 ± 2.6	0.50	21.4 ± 3.2	21.5 ± 2.9	22.2 ± 3.7	0.58
Paternal BMI (kg/m^2^)	24.3 ± 3.1	24.2 ± 2.9	24.3 ± 2.2	0.98	24.5 ± 3.8	25.1 ± 3.0	24.7 ± 3.5	0.77
Hypertensive disorders in pregnancy, Yes	8 (19.5)	5 (13.2%)	5 (14.7)	0.72	2 (6.5%)	2 (5.7%)	5 (12.8%)	0.49
Maternal Secondhand Smoke during Pregnancy, yes	7 (17.1)	9 (23.7)	8 (24.2)	0.69	4 (12.9)	10 (28.6)	12 (30.8)	0.19
Education Level, College or more	40 (97.6)	35 (92.1)	31 (91.2)	0.45	30 (96.8)	31 (88.6)	38 (97.4)	0.32
**Infant Factors**								
Gestational age (weeks)	38.9 ± 1.0	38.9 ± 1.0	38.9 ± 0.9	0.91	38.9 ± 1.0	39.0 ± 1.0	38.6 ± 1.1	0.27
Birthweight (grams)	3500.1 ± 366.9	3551.8 ± 301.2	3454.9 ± 411.8	0.52	3390.0 ± 422.6	3360.9 ± 396.6	3385.0 ± 561.6	0.96
	*n(%*)							
Infant Birthweight for gestational age								
AGA	37 (90.2)	35 (92.1)	28 (82.4)	0.27	26 (83.9)	30 (85.7)	28 (71.8)	0.62
SGA	0 (0.0)	0 (0.0)	2 (5.9)		1 (3.2)	1 (2.9)	2 (5.1)	
LGA	4 (9.8)	3 (7.9)	4 (11.8)		4 (12.9)	4 (11.4)	9 (23.1)	
Child age (months)	23.9 ± 0.5	23.9 ± 0.6	24.0 ± 0.7	0.41	24.1 ± 1.2	24.1 ± 0.8	23.9 ± 0.5	0.67
Infant Feeding in 0–6 months								
Formula feeding	3 (7.7)	3 (9.1)	2 (6.3)	0.60	3 (12.0)	5 (15.6)	4 (12.1)	0.79
Exclusive Breastfeeding	19 (48.7)	14 (42.4)	10 (31.3)		12 (48.0)	13 (40.6)	11 (33.3)	
Mixed	17 (43.6)	16 (48.5)	20 (62.5)		10 (40.0)	14 (43.8)	18 (54.5)	
Child Secondhand Smoke Exposure, Yes	25 (61.0)	22 (57.9)	19 (55.9)	0.90	14 (45.2)	18 (51.4)	19 (50.0)	0.87

**Table 2:** Cardio-metabolic profiles in boys and girls of mothers with prenatal low, medium, and high BPA levels in late pregnancy.

Child cardio-metabolic profiles	Boy			p value	Girl			p value
	Maternal prenatal BPA				Maternal prenatal BPA			
	1st tertile (low)	2nd tertile	3rd tertile (high)		1st tertile (low)	2nd tertile	3rd tertile (high)	
N	41	38	34		31	35	39	
	*mean* ± *SD*				*mean* ± *SD*			
Weight (kg)	13.2 ± 1.2	12.8 ± 1.2	13.0 ± 1.2	0.50	12.5 ± 1.3	12.4 ± 1.1	12.7 ± 1.5	0.65
Length (cm)	89.8 ± 3.2	88.8 ± 3.1	89.8 ± 3.1	0.23	87.5 ± 3.2	88.0 ± 2.8	88.4 ± 3.0	0.48
BMI (kg/m^2^)	16.4 ± 1.0	16.3 ± 1.0	16.2 ± 1.3	0.82	16.4 ± 1.2	16.1 ± 1.1	16.3 ± 1.4	0.66
Weight-for-length z-score	0.42 ± 0.75	0.37 ± 0.76	0.28 ± 0.95	0.76	0.55 ± 0.79	0.37 ± 0.77	0.49 ± 0.93	0.65
Weight-for-age z-score	0.68 ± 0.76	0.46 ± 0.76	0.55 ± 0.80	0.45	0.62 ± 0.81	0.57 ± 0.68	0.75 ± 0.88	0.63
Sum of skinfold thickness (mm)	22.6 ± 4.9	22.5 ± 4.1	22.0 ± 4.3	0.83	23.4 ± 4.5	23.6 ± 4.5	24.2 ± 5.1	0.77
MUAC (cm)	15.8 ± 1.3	15.8 ± 1.1	15.7 ± 1.1	0.77	15.8 ± 1.2	15.8 ± 1.0	15.9 ± 1.3	0.85
SBP (mmHg)	91.8 ± 6.3	91.0 ± 6.3	93.2 ± 8.7	0.48	89.5 ± 6.6	93.1 ± 7.0	95.0 ± 8.7	0.03
DBP (mmHg)	59.7 ± 6.2	60.4 ± 7.8	61.5 ± 6.2	0.57	59.1 ± 3.6	60.8 ± 6.5	62.8 ± 6.2	0.08
Glucose (mmol/L)	5.0 ± 0.6	5.3 ± 0.8	4.9 ± 0.4	0.0495	5.0 ± 0.5	4.7 ± 0.6	5.1 ± 0.6	0.03
log (insulin), (pmol/L)	3.4 ± 0.8	3.4 ± 1.3	3.3 ± 0.7	0.81	3.0 ± 1.0	3.1 ± 0.8	3.5 ± 0.7	0.048
TC (mmol/L)	4.3 ± 0.8	4.1 ± 0.6	4.2 ± 0.7	0.68	4.2 ± 0.8	4.3 ± 0.8	4.2 ± 0.8	0.89
Triglycerides (mmol/L)	1.3 ± 0.9	1.0 ± 0.5	1.2 ± 0.5	0.42	1.0 ± 0.6	1.0 ± 0.5	1.0 ± 0.5	0.94
HDL (mmol/L)	1.4 ± 0.4	1.4 ± 0.3	1.4 ± 0.3	0.97	1.4 ± 0.4	1.3 ± 0.3	1.4 ± 0.3	0.61
LDL (mmol/L)	2.3 ± 0.6	2.2 ± 0.5	2.4 ± 0.4	0.54	2.4 ± 0.6	2.4 ± 0.5	2.4 ± 0.6	0.93

ANOVA was used to test the difference among the three BPA groups. MUAC: mid-upper arm circumference.

## 3 Results

### 3.1 Study population

The study included 218 mother-infant pairs. The mean maternal age at childbirth was 30.6 (SD 3.5) years. Most (94.0%) of the mothers had college education or above. Among the 218 women, 9.2% were overweight (prepregnancy BMI 25–29.9 kg/m^2^), 3 women (1.4%) were obese with prepregnancy BMI ≥30 kg/m^2^, and 13.3% were underweight with a prepregnancy BMI <18.5 kg/m^2^; 1 woman (0.46%) had chronic hypertension, 8.7% (n = 19) had gestational hypertension, and 3.2% (n = 7) had preeclampsia. None of the women had eclampsia. Among the 218 children, 99.1% (n = 216) were term born infants (≥37 weeks), 2.8% were born SGA (n = 6), and 12.8% were born LGA (n = 28). The mean age of children was 24.0 (SD 0.7) months at the follow-up visit. Among 218 children at the age of 2 years, 5 were overweight and 2 were wasted.

Urinary BPA concentration was detectable (>0.1 μg/L) in 98.2% of mothers at late-pregnancy, and in 99.4% of children at 2 years. Median prenatal urinary BPA was 1.14 μg/L (inter quartile range (IQR): 0.63–2.58 μg/L), and median prenatal creatinine-adjusted BPA was 2.21 (IQR 1.35–4.02) (μg/g Cr) in mothers. In children, median creatinine-adjusted urinary BPA was 2.80 (IQR 1.89–5.29) (μg/g Cr) in this study. No sex differences were found in maternal prenatal urinary BPA levels or in children’s BPA levels.

Table 1 presents baseline maternal and infant characteristics by prenatal BPA level (low, medium, and high tertiles) in boys and girls at age of 2 years. There were no differences in maternal education level, maternal passive smoking during pregnancy, infant LGA status, infant passive smoking, and 0–6 month breastfeeding type, infant urinary creatinine-adjusted BPA, and weight-for-length z-score at the age of 2 years among the three prenatal BPA groups (low, medium, and high) in both boys and girls.

### 3.2 Prenatal BPA and cardio-metabolic risk factors in boys and girls

As shown in Table 2, overall, SBP, plasma glucose, and serum insulin were not same among the three prenatal BPA groups (low, medium, and high; ANOVA test, p < 0.05) in girls, nor plasma glucose in boys (ANOVA test, p < 0.05). The SBP and DBP levels increased with higher maternal prenatal urinary BPA levels in girls but not in boys. No differences were found for other cardio-metabolic risk factors among the three prenatal BPA groups in girls. No differences were found in any adiposity measures (weight-for-length z-score, BMI, weight-for-age z-score, sum of skinfold thickness), SBP and DBP, serum lipids (HDL, LDL, TC, and triglycerides), and insulin among the three prenatal BPA groups in boys.

**Fig. 1:**
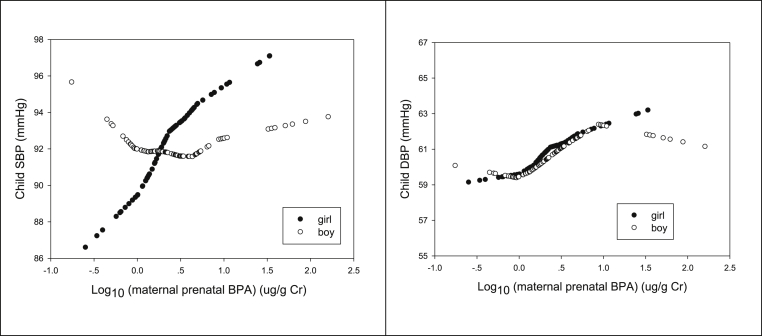
Smoothing scatter plots of child systolic (SBP) and diastolic blood pressure (DBP) (mmHg) by maternal prenatal urinary creatinine-adjusted BPA (μg/g Cr) in base 10 logarithm transformation with adjustment for child age in boys and girls.

We further explored the associations between maternal prenatal BPA and child cardio-metabolic risk factors using a series of regression models with adjustment for potential confounders and covariates in a step by step fashion. Fig. 1, Table 3, Table 4 show the associations between maternal prenatal BPA and blood pressure in girls and boys, respectively.

**Table 3:** The association between maternal prenatal urinary BPA level (μg/L) and blood pressure in 105 girls, Shanghai, China.

Maternal prenatal BPA (μg/L)	Blood Pressure of Girls at Age 2 Years							
	Model 1		Model 2		Model 3		Model 4	
	β (95% CI)	p-value	β (95% CI)	p-value	β (95% CI)	p-value	β (95% CI)	p-value
	SBP (mmHg)							
Low (0.06–0.76)	Ref.		Ref.		Ref.		Ref.	
Medium (0.82–1.91)	4.0 (−0.1,8.2)	0.058	4.4 (0.3,8.4)	0.034	4.7 (1.0,8.4)	0.01	4.6 (0.8,8.3)	0.02
High (1.92–154.6)	6.7 (1.9,11.4)	0.006	6.6 (2.0,11.2)	0.005	7.2 (3.0,11.3)	0.0007	7.0 (2.9,11.2)	0.0009
p-trend		0.007		0.006		0.0008		0.001
Log_10_BPA (μg/L)	5.6 (1.6, 9.6)	0.006	5.6 (1.7, 9.4)	0.005	4.8 (1.2, 8.4)	0.009	5.2 (1.5, 8.8)	0.005
	**DBP (mmHg)**							
Low (0.06–0.76)	Ref.		Ref.		Ref.		Ref.	
Medium (0.82–1.91)	1.6 (−1.6,4.8)	0.32	1.7 (−1.5,4.8)	0.30	1.8 (−1.1,4.7)	0.23	2.5 (−0.3,5.4)	0.082
High (1.92–154.6)	3.8 (0.1,7.4)	0.045	3.7 (0.1,7.4)	0.047	4.0 (0.7,7.3)	0.02	4.4 (1.2,7.5)	0.007
p-trend		0.04		0.045		0.02		0.008
Log_10_BPA (μg/L)	2.3 (−0.8, 5.4)	0.14	2.3 (−0.8, 5.4)	0.14	1.6 (−1.2, 4.4)	0.26	2.3(-0.5, 5.1)	0.11

Model 1: adjusted for log (maternal urinary creatinine) and child age.

Model 2: adjusted for log (maternal urinary creatinine), LGA status (LGA, non-LGA), and child age.

Model 3: adjusted for log (maternal urinary creatinine), LGA status (LGA, non-LGA), children’s age, urinary creatinine-adjusted BPA tertiles (low, medium and high), and weight-for-length z-score.

Model 4: adjusted for log (maternal urinary creatinine), maternal passive smoking (yes/no), hypertensive disorders in pregnancy (yes/no), infant LGA status (LGA, non-LGA), children’s age, passive smoking (yes/no), child urinary creatinine-adjusted BPA tertiles (low, medium and high), 0–6 months breastfeeding type (Formula feeding, Exclusive breastfeeding, and Mixed feeding), and weight-for-length z-score.

**Table 4:** The association between maternal prenatal urinary BPA level (μg/L) and blood pressure in 113 boys, Shanghai, China.

Maternal prenatal BPA (μg/L)	Blood Pressure of Boys at Age 2 Years							
	Model 1		Model 2		Model 3		Model 4	
	β (95% CI)	p-value	β (95% CI)	p-value	β (95% CI)	p-value	β (95% CI)	p-value
	SBP (mmHg)							
Low (0.07–0.74)	Ref.		Ref.		Ref.		Ref.	
Medium (0.82–1.84)	−0.8 (−4.4,2.7)	0.64	−1.0 (−4.6,2.6)	0.59	−1.6 (−5.0,1.8)	0.36	−1.3 (−4.7,2.1)	0.45
High (1.99–105.13)	1.4 (−2.5,5.4)	0.48	1.3 (−2.6,5.3)	0.50	1.0 (−2.8,4.7)	0.61	1.5 (−2.3,5.2)	0.44
p-trend		0.47		0.48		0.58		0.41
Log_10_BPA (μg/L)	−0.1 (−2.9, 2.7)	0.97	−0.1 (−2.9, 2.7)	0.92	0.2 (−2.4, 2.9)	0.88	0.3 (−2.3, 2.9)	0.83
	**DBP (mmHg)**							
Low (0.07–0.74)	Ref.		Ref.		Ref.		Ref.	
Medium (0.82–1.84)	1.7 (−1.8,5.2)	0.34	1.3 (−2.1,4.8)	0.45	1.5 (−1.9,4.9)	0.40	1.8 (−1.5,5.2)	0.28
High (1.99–105.13)	3.3 (−0.5,7.2)	0.09	3.2 (−0.6,6.9)	0.10	3.2 (−0.4,6.9)	0.08	4.0 (0.3,7.6)	0.03
p-trend		0.090		0.10		0.08		0.03
Log_10_BPA (μg/L)	1.1 (−1.6, 3.8)	0.41	0.9 (−1.7, 3.6)	0.50	1.2 (−1.4, 3.8)	0.38	1.3 (−1.3, 3.8)	0.33

Model 1: adjusted for log (maternal urinary creatinine) and child age.

Model 2: adjusted for log (maternal urinary creatinine), LGA status (LGA, non-LGA), and child age.

Model 3: adjusted for log (maternal urinary creatinine), LGA status (LGA, non-LGA), children’s age, urinary creatinine-adjusted BPA tertiles (low, medium and high), and weight-for-length z-score.

Model 4: adjusted for log (maternal urinary creatinine), maternal passive smoking (yes/no), hypertensive disorders in pregnancy (yes/no), infant LGA status (LGA, non-LGA), children’s age, passive smoking (yes/no), urinary creatinine-adjusted BPA tertiles (low, medium and high), 0–6 months breastfeeding type (Formula feeding, Exclusive breastfeeding, and Mixed feeding), and weight-for-length z-score.

As shown in Table 3, compared to those with low prenatal BPA, mean SBP was 4.6 (95%CI: 0.8–8.3) mmHg higher in girls with the medium prenatal BPA, and 7.0 (95%CI: 2.9–11.2) mmHg higher in girls with the high prenatal BPA with adjustment for current weight-for-length z-score, child urinary BPA, and other covariates (Model 4, p_trend_ = 0.001). In girls, each 10-fold increase in maternal prenatal BPA was associated with 5.2 mmHg (95%CI: 1.5–8.8 mmHg) increased SBP. To a lesser extent, DBP was 4.4 mmHg (95%CI: 1.2–7.5 mmHg) higher in girls with high prenatal BPA compared to those with the low prenatal BPA (Table 3). No associations were observed between maternal prenatal BPA and SBP or DBP in boys (Table 4，Fig. 1).

As shown in Table 5, plasma glucose appeared to be 0.26 mmol/L lower in girls with medium prenatal BPA level, but the difference was not statistically significant (95% CI -0.55 to 0.04 mmol/L, p = 0.08; Model 4). In contrast, medium prenatal BPA level was associated with 0.36 (95% CI: 0.04, 0.68) mmol/L higher plasma glucose with adjustment for current weight-for-length z-score and other covariates in boys at age 2 years (Table 6, Model 4).

**Table 5:** The association between maternal prenatal urinary BPA level (μg/L) and blood glucose and insulin in 105 girls, Shanghai, China.

Maternal prenatal BPA (μg/L)	Plasma Glucose and Serum Insulin of Girls at Age 2 Years							
	Model 1		Model 2		Model 3		Model 4	
	β (95% CI)	p-value	β (95% CI)	p-value	β (95% CI)	p-value	β (95% CI)	p-value
	Glucose (mmol/L)							
Low	Ref.		Ref.		Ref.		Ref.	
Medium	−0.27 (−0.57,0.03)	0.07	−0.27 (−0.57,0.03)	0.08	−0.24 (−0.55,0.06)	0.11	−0.26 (−0.55,0.04)	0.08
High	0.14 (−0.18,0.45)	0.40	0.14 (−0.18,0.45)	0.40	0.15 (−0.16,0.46)	0.35	0.08 (−0.22,0.38)	0.61
Log_10_BPA (μg/L)	0.24 (0.02,0.46)	0.04	0.24 (0.02,0.47)	0.03	0.24 (0.02,0.46)	0.04	0.17 (−0.04,0.39)	0.12
	**Natural log (insulin) (pmol/L)**							
Low	Ref.		Ref.		Ref.		Ref.	
Medium	0.02 (−0.42,0.47)	0.92	0.03 (−0.42,0.47)	0.90	0.09 (−0.36,0.54)	0.70	−0.01 (−0.44,0.41)	0.95
High	0.33 (−0.14,0.80)	0.17	0.33 (−0.14,0.80)	0.16	0.35 (−0.11,0.81)	0.14	0.23 (−0.20,0.67)	0.29
Log_10_BPA (μg/L)	0.27 (−0.06,0.59)	0.11	0.27 (−0.06,0.60)	0.11	0.26 (−0.06,0.58)	0.11	0.19 (−0.12,0.50)	0.23

Model 1: adjusted for log (maternal urinary creatinine) and child age.

Model 2: adjusted for log (maternal urinary creatinine), LGA status (LGA, non-LGA), and child age.

Model 3: adjusted for log (maternal urinary creatinine), LGA status (LGA, non-LGA), children’s age, urinary creatinine-adjusted BPA tertiles (low, medium and high), and weight-for-length z-score.

Model 4: adjusted for log (maternal urinary creatinine), maternal passive smoking (yes/no), hypertensive disorders in pregnancy (yes/no), infant LGA status (LGA, non-LGA), children’s age, passive smoking (yes/no), child urinary creatinine-adjusted BPA tertiles (low, medium and high), 0–6 months breastfeeding type (Formula feeding, Exclusive breastfeeding, and Mixed feeding), and weight-for-length z-score.

**Table 6:** The association between maternal prenatal urinary BPA level (μg/L) and blood glucose and insulin in 113 boys, Shanghai, China.

Maternal prenatal BPA (μg/L)	Plasma Glucose and Serum Insulin of Boys at Age 2 Years							
	Model 1		Model 2		Model 3		Model 4	
	β (95% CI)	p-value	β (95% CI)	p-value	β (95% CI)	p-value	β (95% CI)	p-value
	Glucose (mmol/L)							
Low	Ref.		Ref.		Ref.		Ref.	
Medium	0.34 (0.02,0.66)	0.04	0.29 (−0.03,0.61)	0.07	0.31 (−0.01,0.64)	0.06	0.36 (0.04,0.68)	0.03
High	−0.11 (−0.49,0.26)	0.56	−0.15 (−0.52,0.22)	0.44	−0.15 (−0.52,0.22)	0.44	−0.06 (−0.44,0.31)	0.74
Log_10_BPA (μg/L)	0.02 (−0.26,0.30)	0.90	−0.01 (−0.29,0.27)	0.95	−0.01 (−0.30,0.27)	0.92	0.02 (−0.27,0.30)	0.92
	**Natural log (insulin) (pmol/L)**							
Low	Ref.		Ref.		Ref.		Ref.	
Medium	−0.08 (−0.59,0.42)	0.75	−0.12 (−0.63,0.40)	0.66	−0.08 (−0.58,0.42)	0.75	−0.07 (−0.57,0.43)	0.78
High	−0.34 (−0.93,0.26)	0.27	−0.36 (−0.95,0.24)	0.24	−0.34 (−0.92,0.23)	0.24	−0.37 (−0.94,0.21)	0.22
Log_10_BPA (μg/L)	−0.16 (−0.59,0.27)	0.46	−0.18 (−0.61,0.26)	0.43	−0.14 (−0.57,0.28)	0.50	−0.17 (−0.59,0.25)	0.43

Model 1: adjusted for log (maternal urinary creatinine) and child age.

Model 2: adjusted for log (maternal urinary creatinine), LGA status (LGA, non-LGA), and child age.

Model 3: adjusted for log (maternal urinary creatinine), LGA status (LGA, non-LGA), children’s age, urinary creatinine-adjusted BPA tertiles (low, medium and high), and weight-for-length z-score.

Model 4: adjusted for log (maternal urinary creatinine), maternal passive smoking (yes/no), hypertensive disorders in pregnancy (yes/no), infant LGA status (LGA, non-LGA), children’s age, passive smoking (yes/no), child urinary creatinine-adjusted BPA tertiles (low, medium and high), 0–6 months breastfeeding type (Formula feeding, Exclusive breastfeeding, and Mixed feeding), and weight-for-length z-score.

No associations were observed between prenatal BPA level and child weight-for-length z-score, BMI, weight-for-age z-score, skinfold thicknesses, insulin, HDL, LDL, cholesterol, triglycerides or DBP in either girls or boys (Table 2, Table 5, Table 6). Children’s current urinary BPA concentrations were not associated with these cardio-metabolic risk factors in boys and girls (data not shown).

## 4 Discussion

In this prospective birth cohort, we examined the association between maternal prenatal BPA level and a wide range of cardio-metabolic risk factors in children at the age of 2 years. We found that high prenatal urinary BPA concentration was associated with higher SBP and DBP in girls, but not in boys. Plasma glucose was higher in boys with medium maternal prenatal BPA level. No associations were observed between maternal prenatal BPA level and adiposity measures, serum insulin, or serum lipid profiles in the children. Our findings implicate early exposure to environmental chemicals as another “novel” risk factor for high BP and impaired glucose tolerance in later life.

The findings from the present study support a sex-specific and positive association between prenatal BPA exposure and child BP, and a curve association between prenatal BPA exposure and plasma glucose in children. The fetal period is a critical window in susceptibility to the potential impact of environmental factors on cardiovascular system (Mone et al., 2004). In the fetus, BPA exposure originates transplacental from the pregnant mother. However, limited epidemiologic data exists to connect prenatal BPA exposure and cardio-metabolic risk factors of children, as the results from these few studies were inconsistent (Bae et al., 2017; Vafeiadi et al., 2016; Warembourg et al., 2019). Previous studies reported that prenatal BPA was associated with child higher DBP (Warembourg et al., 2019), showed no associations (Vafeiadi et al., 2016), or was associated with DBP in boys and SBP in girls under the condition of prenatal urinary BPA concentrations above a certain threshold (Bae et al., 2017). A pooled analysis of the European HELIX (Human Early-Life Exposome) data showed that prenatal BPA exposure was associated with 0.7 mmHg higher child DBP (95%CI: 0.1–1.4 mmHg) per an interquartile-range (4.9 mg/g creatinine) increase in maternal urinary BPA concentrations (Warembourg et al., 2019), while child current urinary BPA was not associated with BP among children at the age of 6–11 years (Warembourg et al., 2019). However, the prospective cohort study in Greece showed that prenatal urinary BPA concentration (which was measured at the ﬁrst trimester) was not associated with either BP or serum cholesterol in boys or girls at age 4 years (Vafeiadi et al., 2016). In a Korean study, maternal prenatal urinary BPA (measured at 20 gestational weeks) was associated with 9.8 mmHg higher child DBP per each log unit of prenatal BPA (μg/g creatinine) in boys given maternal BPA > 5.5 μg/g creatinine; and with 2.1 mmHg higher SBP in girls given maternal BPA above a threshold of 0.7 μg/g creatinine, and a negative association with child SBP if maternal BPA below the threshold (Bae et al., 2017). In the present study, although prenatal BPA was marginally associated with boys’ DBP, the magnitude and significance of the prenatal BPA association with both SBP and DBP were greater in girls. As for child current urinary BPA, it was not associated with blood pressure or with most other metabolic cardiovascular risk factors in children aged 4–11 years in previous studies (Bae et al., 2017; Vafeiadi et al., 2016; Warembourg et al., 2019) or children aged 2 years in the present study. Thus, the association between prenatal BPA and child BP in this study is less likely to be explained by child current BPA level, child adiposity measures and other metabolic risk factors.

In this study, neither prenatal nor child BPA was associated with adiposity measures in children at the age of 2 years. Consistently, prenatal urinary BPA were not associated with child anthropometric adiposity measures in the children of 2–5 years in the Health Outcomes and Measures of the Environment (HOME) Study conducted in the U.S. (Braun et al., 2014), or children 8–14 years of old in the Early Life Exposure in Mexico to Environmental Toxicants (ELEMENT) study (Yang et al., 2017), boys aged 7 years in the Columbia Center for Children’s Environmental Health (CCCEH) birth cohort in New York city (Hoepner et al., 2016), and boys of 9 years in the Center for the Health Assessment of Mothers and Children of Salinas (CHAMACOS) (Harley et al., 2013). However, prenatal urinary BPA was found to be negatively associated with BMI in girls at age 3–4 years (sex interaction test, p-value < 0.05) in a Greek study (Vafeiadi et al., 2016) and in girls at the age of 9 years in the CHAMACOS cohort (Harley et al., 2013). In contrast, prenatal BPA was positively associated with BMI in boys at age of 3–4 years (sex interaction p-value <0.05) in the same Greek study (Vafeiadi et al., 2016), and with fat mass index (FMI) and waist circumference in girls aged 7 years in the in the CCCEH cohort study (Hoepner et al., 2016). As for the associations between child BPA and adiposity measures, previous studies produced inconsistent results. Child urinary BPA was not associated with child anthropometric measures in children at age 2.5 years in the Greek study (Vafeiadi et al., 2016), age 2–5 years in the HOME study (Braun et al., 2014), or age 7 years in the CCCEH study (Hoepner et al., 2016). A positive association of child BPA with adiposity measures was observed in both boys and girls at age 4 years in the Greek study (Vafeiadi et al., 2016), at age 9 years in the CHAMACOS cohort (Harley et al., 2013), and, with skinfold thickness among girls but not boys, at age 8–14 years in the ELEMENT study (Yang et al., 2017). The present study together with other studies suggest that associations between child current BPA and adiposity measures might differ by timing of the BPA exposure (prenatal or postnatal), child age and sex (Yang et al., 2017).

Cross-sectional analysis between BPA and cardiovascular risk factors have been largely conducted in adults or older children (Aekplakorn et al., 2015). A recent Iran study showed that urinary BPA was associated with higher BMI, SBP and DBP in children age 6–18 years (BPA geometric mean, 232.6 μg/L) (Amin et al., 2019). On the other hand, a small study of overweight/obese children (n = 39) in the U.S. found that urinary BPA was linked with elevated DBP in boys 3–8 years old, but not in girls of the same age (Khalil et al., 2014). In a randomized crossover trial of adults (age ≥60 years), after acute exposure to BPA by drinking two canned beverages versus the control by drinking two glass-bottled beverages, SBP consequently increased (by around 4.5 mmHg) with the increase in urinary BPA concentration (Mean ± SD, 1.13 ± 1.76 versus 16.91 ± 12.55 μg/L) (Bae and Hong, 2015). A cross sectional study also showed an association between urinary BPA concentration and hypertension, independent of BMI and diabetes in adults (Shankar and Teppala, 2012). To better understand the long-term effects of prenatal BPA exposure on cardiovascular diseases or their precursors, cohort studies with longer follow-up and larger sample sizes are needed.

The finding in the present study is biologically plausible. Animal studies have reported that prenatal BPA exposure (during the second half of pregnancy) results in increased SBP in mice offspring even long after BPA, which suggested that fetal exposure to BPA during its development can produce persistent effects on future hypertension (Cagampang et al., 2012) which might be conferred through a multifactorial mechanism. The adverse effects of BPA on renal development might be one mechanism in the programing of adult BP (Cagampang et al., 2012; Langley-Evans et al., 2003). Animal studies have reported that BPA exposure during embryonic development alters nephrogenesis, and causes reduced nephron quantity, lower glomeruli density and renal structural change in female offspring of BPA-treated mice dams (Nunez et al., 2018). In addition, female mice who were developmental intrauterine exposure to BPA had altered energy metabolism of carbohydrates/fats, decreased motivation for physical activity and were less active (Johnson et al., 2015). All these changes might increase risk of offspring developing cardiovascular and metabolic dysfunction later in life (Johnson et al., 2015; Nunez et al., 2018). In humans, nephrogenesis begins at 9 gestational weeks, and achieved the complement of ∼1 million nephrons by ∼35 gestational weeks (Sanders et al., 2018). As nephron number does not change with age, environmental factors with the impact on nephrons number reduce in fetal kidney during the process of nephrogenesis, can lead to higher risk of future hypertension (Briffa et al., 2018). Future studies on the mechanism underlying prenatal BPA associated offspring hypertension are needed. The first 1000 days of life is a critical opportunity window for an intervention strategy to modify this programming and reduce the risk of cardiometabolic disorders (Lurbe et al., 2016). Our study findings highlight the importance of environmental chemical intrauterine exposures like BPA in the programming of elevated BP later in life.

Child hypertension is a considerable global public health challenge (Song et al., 2019). Its prevalence throughout the world increased by more than 75% among children aged 6–19 years in 2000–2015 (Song et al., 2019). The overall prevalence of hypertension is about 28.9% in China (Wang et al., 2016). Early life BP elevation is strongly correlated with later hypertension(Sanders et al., 2018), which is a strong predictor of other cardiovascular disease including stroke. BPA’s effect on BP is of particular interest for the Chinese population. China has an increased incidence of hypertension (28.9%), despite a significantly lower rate of obesity compared to western countries (Wang et al., 2016). A better understanding of BPA’s association with hypertension may further help explain the high incidence of hypertension at all ages.

The primary strengths of the present study are that we have collected data on multiple pre-, peri- and postnatal risk factors, and a wide range of meta-cardiovascular risk factor in children, an understudied age group. The study also had limitations. We collected a one-time measurement of prenatal urinary BPA concentration in the third trimester . However, previous studies showed that the categories of urinary BPA concentration in spot urine samples by tertiles (low, medium and high) can reflect individual chronic exposure levels of BPA over weeks to months in epidemiologic population studies (Mahalingaiah et al., 2008; Teitelbaum et al., 2008). BPA can rapidly cross placenta and accumulate in the fetus, with fetal BPA exceeding maternal levels after even an acute single maternal exposure to BPA (Takahashi and Oishi, 2000). Fasting blood sample collection is challenging in children of 2 years, so caution is need when interpreting plasma glucose and serum triglycerides levels of children in this study. This study only followed the children up to 2 years, and long-term follow-up will allow us to assess long-term health impact. Finally, this study may have limited power due to its sample size. Further independent cohort studies in women with high urinary BPA are needed to confirm the findings.

## 5 Conclusion

In this Chinese prospective birth cohort study, we found that maternal prenatal urinary BPA concentration was associated with higher SBP and DBP in girls, but not in boys, independent of adiposity measures. The medium prenatal BPA level was associated with higher plasma glucose in boys. No associations were observed between child urinary BPA concentration and cardio-metabolic risk factors. Further independent cohort studies are needed to confirm the findings.

## CRediT authorship contribution statement

**Fengxiu Ouyang:** Conceptualization, Investigation, Formal analysis, Writing - original draft, Writing - review & editing, Funding acquisition. **Guang-Hui Zhang:** Methodology. **Kun Du:** Methodology. **Lixiao Shen:** Investigation. **Rui Ma:** Investigation. **Xia Wang:** Methodology, Investigation. **Xiaobin Wang:** Writing - review & editing. **Jun Zhang:** Investigation, Funding acquisition, Writing - review & editing.

## Declaration of competing interest

None of the authors have a conflict of interest pertaining to this work.
